# Anticoagulation after Anterior Myocardial Infarction and the Risk of Stroke

**DOI:** 10.1371/journal.pone.0012150

**Published:** 2010-08-13

**Authors:** Jacob A. Udell, Julie T. Wang, David J. Gladstone, Jack V. Tu

**Affiliations:** 1 Institute for Clinical Evaluative Sciences, Toronto, Ontario, Canada; 2 Department of Medicine, Sunnybrook Health Sciences Centre, University of Toronto, Toronto, Ontario, Canada; 3 Division of Cardiovascular Medicine, Department of Medicine, Brigham and Women's Hospital, Boston, Massachusetts, United States of America; New York University School of Medicine, United States of America

## Abstract

**Background:**

Survivors of anterior MI are at increased risk for stroke with predilection to form ventricular thrombus. Commonly patients are discharged on dual antiplatelet therapy. Given the frequency of early coronary reperfusion and risk of bleeding, it remains uncertain whether anticoagulation offers additional utility. We examined the effectiveness of anticoagulation therapy for the prevention of stroke after anterior MI.

**Methods and Findings:**

We performed a population-based cohort analysis of 10,383 patients who survived hospitalization for an acute MI in Ontario, Canada from April 1, 1999 to March 31, 2001. The primary outcome was four-year ischemic stroke rates compared between anterior and non-anterior MI patients. Risk factors for stroke were assessed by multivariate Cox proportional-hazards analysis. Warfarin use was determined at discharge and followed for 90 days among a subset of patients aged 66 and older (n = 1483). Among the 10,383 patients studied, 2,942 patients survived hospitalization for an anterior MI and 20% were discharged on anticoagulation therapy. Within 4 years, 169 patients (5.7%) were admitted with an ischemic stroke, half of which occurred within 1-year post-MI. There was no significant difference in stroke rate between anterior and non-anterior MI patients. The use of warfarin up to 90 days was not associated with stroke protection after anterior MI (hazard ratio [HR], 0.68; 95% confidence interval [CI], 0.37–1.26). The use of angiotensin-converting-enzyme inhibitors (HR, 0.65; 95% CI, 0.44–0.95) and beta-blockers (HR, 0.60; 95% CI, 0.41–0.87) were associated with a significant decrease in stroke risk. There was no significant difference in bleeding-related hospitalizations in patients who used warfarin for up to 90 days post-MI.

**Conclusion:**

Many practitioners still consider a large anterior-wall MI as high risk for potential LV thrombus formation and stroke. Among a cohort of elderly patients who survived an anterior MI there was no benefit from the use of warfarin up to 90 days post-MI to prevent ischemic stroke. Our data suggests that routine anticoagulation of patients with anterior-wall MI may not be indicated. Prospective randomized trials are needed to determine the optimal antithrombin strategy for preventing this common and serious adverse outcome.

## Introduction

Patients with acute ST-segment elevation myocardial infarction (STEMI) have an elevated risk of stroke, most of which are ischemic in origin [1]. The risk for stroke after myocardial infarction (MI) is estimated to be 44-fold higher within the first 30 days, and remains 2 to 3 times higher than expected during the subsequent 3 years [1]. Longitudinal stroke risk following an MI is estimated to be approximately 1 percent by the first month, 2 percent by one year, and 5 percent by four years [2], [3], [4].

The association between the size, severity, and location of an MI and risk of developing stroke remains controversial [5]–[14]; nonetheless, practice guidelines recommend anticoagulation in certain settings [15]. For instance, left ventricular (LV) thrombus formation after an MI poses an increased risk of cardioembolism, which is reduced by anticoagulation [7], [14], [16]–[25]. Anterior-wall location of a MI has historically been considered a surrogate marker for potential focal dyskinesia leading to LV aneurysm or thrombus complication, which some estimate occurs in approximately one-third of individuals within the first 2 weeks following an anterior MI [26]. Myocardial infarction treatment patterns and subsequent post-MI complications have evolved dramatically in the past 20 years, particularly with regard to effectiveness and expediency in medication use, revascularization, ventricular imaging, and hospital discharge. As a result, practice patterns vary on whether or not anterior MI alone warrants anticoagulation in an era of early revascularization and coronary artery stent therapy that may reduce LV dysfunction or LV thrombus formation.

The devastating impact of a stroke after an MI, and the increasing number of persons at risk because of improved post-MI survival, constitutes an important public health matter for persons with heart disease. Consequently, the effectiveness of anticoagulation therapy after anterior MI for the prevention of stroke warrants further investigation.

## Methods

### Study Population

The design of the Enhanced Feedback for Effective Cardiac Treatment (EFFECT) study has been described previously [27], [28]. The EFFECT study is a large province-wide initiative designed to improve the quality of acute MI care in Ontario, Canada. In summary, the EFFECT database consists of a large population-based sample of acute MI patients hospitalized throughout Ontario, Canada between April 1, 1999 and March 31, 2001. The hospitals included university-affiliated and community-based institutions from both rural and urban settings. All had admitted more than 30 patients with acute MI during the two years of sampling.

For this study, we excluded patients who had a previously recorded MI admission within the past year, those who sustained a MI as an in-hospital complication (e.g. post-operative), those who were transferred from an outside institution, those younger than 20 or 105 years of age or older, nonresidents of the province of Ontario, and those with invalid health insurance numbers. We also excluded those with a history of warfarin use due to previous atrial fibrillation, valvular disease, carotid endarterectomy, or thromboembolic disease, because these patients may have had other indications (other than an anterior MI) for receiving warfarin post infarction.

For the diagnosis of an acute MI, each patient had to meet the European Society of Cardiology/American College of Cardiology clinical criteria for acute MI [29], and its onset must have occurred before the patient arrived at the hospital.

The records of patients who met the inclusion and exclusion criteria for the study were reviewed by trained cardiology research nurses for abstraction of clinical data and processes of care related to the index MI hospitalization. Random re-abstraction of charts indicated high interrater agreement [30]. A random sample of 125 patients was identified among hospitals treating at least 125 suitable candidates during the study period. For hospitals that treated fewer than 125 suitable candidates during the study period, all charts were reviewed. Among the 104 acute care hospitals eligible for the study, 103 institutions ultimately participated in the study. A total of 11 524 patients with a most-responsible diagnosis of AMI (*International Classification of Diseases*, *Ninth Revision*, *Clinical Modification* [ICD-9] code 410) were identified using the Canadian Institutes of Health Information (CIHI) hospital discharge abstract database [27], [28]. Additional details related to the exclusion/inclusion criteria and validation of the AMI coding have been published elsewhere [27], [28], [31]–[33]. Administrative data were linked anonymously using encrypted individual health card numbers.

### Ethics Statement

The study protocol was submitted to and approved by the research ethics boards at the participating institutions and Sunnybrook Health Sciences Centre. The research ethics boards approved a waiver of informed consent for collecting the study data due to the minimal risk nature of the study [34], [35].

### Definitions, Study Outcomes and Data Sources

We defined a set of clinical and demographic variables that may potentially be associated with stroke after MI that were available from chart review. Patient hospitalizations were identified using ICD-9 and 10 codes in CIHI hospital discharge abstracts to longitudinally characterize subsequent events and co-morbid illnesses (Table S1). We used each patient's encrypted identification number to link hospital discharge data to the Registered Persons Database (RPDB) in order to calculate mortality. The RPDB provides data on the vital status of residents of Ontario and records both in- and out-of-hospital death. The Ontario Drug Benefits (ODB) database was used to longitudinally identify medications each participant aged 66 and older was dispensed during the observation period, from Ontario's universal drug benefits plan.

The primary study cohort was those patients who were diagnosed as having an acute anterior-wall MI, with additional analyses conducted using the complete data set of all MI survivors. Anterior location of the MI was defined as a diagnostic electrocardiogram having either Q-waves or ST-elevation greater than 1 mm in leads I and aVL or V_1_ through V_4_. A subset of the above cohort (n = 1483), those aged 66 and older, was selected to analyze the effect of out-patient medication use on outcomes.

The primary study outcome was the rate of ischemic stroke (Table S1) among acute anterior-wall MI patients who survived to hospital discharge. Secondary outcomes focused on death from any cause and bleeding-related complications, such as hospitalization for gastrointestinal (GI) or cerebral hemorrhage. We evaluated warfarin use among the entire cohort upon discharge, and continued warfarin use 30, 60, and 90 days after the index MI among those aged 66 and older, as the exposure of interest. We chose to study warfarin use at monthly intervals between discharge and 90 days in order to determine whether there is a significant protective effect of longitudinal warfarin use on the rate of stroke in routine clinical practice among those who take warfarin for any duration, including up to the guideline-recommended three months.

### Statistical Analysis

The demographic and clinical characteristics of patients with anterior MI and other types of MI were compared. Dichotomous variables were compared by the chi-squared test and continuous variables by the Student's t-test.

We used Cox proportional-hazards analysis to identify factors associated with an increased risk of ischemic stroke after hospitalization for an anterior MI. Candidate variables were included in the initial Cox regression model if they were associated with stroke in a univariate analysis (P<0.25). Backward variable elimination, with an elimination criterion of a P-value of more than 0.05, was then used to create a parsimonious model for predicting ischemic stroke. We determined that the assumption of proportional hazards was met in all Cox regression models. Survival-free of stroke curves were constructed for warfarin users and non-users among anterior-wall MI patients.

The results are shown as means ± SD unless otherwise indicated. Statistical analysis was performed using SAS version 9.1 (SAS Institute Inc, Cary, NC); a P-value less than 0.05 was considered statistically significant.

## Results

### Clinical Characteristics

Among the 11 524 index MI patients assessed for eligibility for this study, 10 383 patients survived to hospital discharge, of which 2 942 (28%) had an anterior-wall MI (50% or 1483 were patients aged 66 or older). The clinical characteristics of the study patients are shown in Table 1. Patients who survived an anterior MI to discharge were younger (65 vs. 67 years, P<0.001), more likely to be male (67 percent vs. 65 percent P = 0.028), and had a significantly lower incidence of previous MI (20 percent vs. 23 percent P<0.001) as compared to those who survived other types of MI. Anterior MI patients were more likely to present with significant left ventricular dysfunction (defined as an LV ejection fraction <40% or grade 3 or 4, or narrative description of LV function as “moderate” or “severe”) (23 percent vs. 11 percent P<0.001) and had a higher likelihood of developing a left ventricular aneurysm, thrombus or ventricular septal defect (5 percent vs. 1 percent P<0.001) as a complication of the index MI. Upon discharge from hospital, anterior MI patients were more likely to be prescribed an anticoagulant (22 percent vs. 12 percent; P<0.001), including warfarin (14 percent vs. 6 percent; P<0.001), an angiotensin-converting-enzyme (ACE) inhibitor (59 percent vs. 52 percent; P<0.001) and clopidogrel (6 percent vs. 5 percent; P = 0.02), but less likely to be prescribed aspirin (73 percent vs. 77 percent; P<0.001), calcium antagonists (22 percent vs. 28 percent; P<0.001), and nitrates (38 percent vs. 44 percent; P<0.001).

**Table 1:** Characteristics of 10,383 Myocardial Infarction Patients Discharged Alive.

Variable	Total	Anterior MI	Other MI	P-value
	N = 10,383	N = 2,942	N = 7,441	
**Patient Demographics**				
Age	66.46±13.51	65.52±13.75	66.84±13.39	<.001
Male	6,805 (65.5%)	1,976 (67.2%)	4,829 (64.9%)	0.03
**Past Medical History**				
Diabetes mellitus	2,621 (25.2%)	707 (24.0%)	1,914 (25.7%)	0.07
Myocardial infarction	2,333 (22.5%)	595 (20.2%)	1,738 (23.4%)	<.001
Congestive heart failure	418 (4.0%)	120 (4.1%)	298 (4.0%)	0.86
Hypertension	4,692 (45.2%)	1,291 (43.9%)	3,401 (45.7%)	0.09
Previous smoker	2,569 (24.7%)	674 (22.9%)	1,895 (25.5%)	0.007
Previous atrial fibrillation	122 (1.2%)	26 (0.9%)	96 (1.3%)	0.08
Previous ischemic stroke	133 (1.3%)	33 (1.1%)	100 (1.3%)	0.36
**In Hospital Findings & Procedures**				
Peak creatine kinase (CK) >2 times the upper limit of normal	6,293 (60.6%)	1,966 (66.8%)	4,327 (58.2%)	<.001
Killip class ≥3	248 (2.4%)	82 (2.8%)	166 (2.2%)	0.09
LV dysfunction	1,461 (14.1%)	679 (23.1%)	782 (10.5%)	<.001
LV aneurysm, thrombus, or ventricular septal defect (VSD)	226 (2.2%)	139 (4.7%)	87 (1.2%)	<.001
ST elevation on any lead groups	4,979 (48.0%)	2,474 (84.1%)	2,505 (33.7%)	<.001
Reperfusion therapy	3,414 (32.9%)	1,419 (48.2%)	1,995 (26.8%)	<.001
Discharge systolic blood pressure	120.42±19.68	118.01±19.39	121.36±19.65	<.001
Discharge diastolic blood pressure	67.89±11.62	67.33±12.07	68.11±11.43	0.002
In-hospital atrial fibrillation	620 (6.0%)	171 (5.8%)	449 (6.0%)	0.67
In-hospital ischemic stroke	20 (0.2%)	7 (0.2%)	13 (0.2%)	0.51
**Discharge Medications**				
ACE inhibitors	5,578 (53.7%)	1,745 (59.3%)	3,833 (51.5%)	<.001
Angiotensin II receptor blocker (ARB)	216 (2.1%)	67 (2.3%)	149 (2.0%)	0.38
Antiarrhythmics or digoxin	1,232 (11.9%)	364 (12.4%)	868 (11.7%)	0.32
Anticoagulants	1558 (15.0%)	640 (21.8%)	918 (12.3%)	<.001
Aspirin	7,877 (75.9%)	2,137 (72.6%)	5,740 (77.1%)	<.001
Beta-adrenergic antagonists	7,021 (67.6%)	2,026 (68.9%)	4,995 (67.1%)	0.09
Calcium antagonists	2,713 (26.1%)	645 (21.9%)	2,068 (27.8%)	<.001
Clopidogrel	529 (5.1%)	173 (5.9%)	356 (4.8%)	0.02
Diuretics	2,593 (25.0%)	725 (24.6%)	1,868 (25.1%)	0.63
Low molecular weight heparin (LMWH)	237 (2.3%)	60 (2.0%)	177 (2.4%)	0.30
Nitrates	4,346 (41.9%)	1,102 (37.5%)	3,244 (43.6%)	<.001
Statins	3,453 (33.3%)	952 (32.4%)	2,501 (33.6%)	0.22
Warfarin	870 (8.4%)	400 (13.6%)	470 (6.3%)	<.001

Data are reported as number (percentage) of patients or mean ± standard deviation (SD) value unless otherwise specified.

### Total Incidence of Stroke and Secondary Outcomes

The unadjusted rates of stroke, bleeding, and mortality among the entire cohort are summarized in Table 2. One hundred and seventy-six (6 percent) of the 2 942 patients discharged alive after an anterior-wall MI were diagnosed within 4 years with an acute ischemic stroke (Table 2). Seven strokes (0.2 percent) occurred in-hospital, leaving 169 (5.7 percent) stroke events following hospital discharge, approximately half of which occurred within the first year of discharge. In univariate analysis, there were no significant differences between anterior-wall MI patients and all other MI patients with regard to one- and four-year ischemic stroke rates, as well as the secondary outcomes of in-hospital, 30-day, 90-day stroke rate, all-cause mortality, and hospitalization for bleeding-related complications after one year. These results were similar after excluding those patients who developed in-hospital atrial fibrillation after the index MI. Among the 557 elderly patients who used warfarin for one year, there also was no significant difference in bleeding-related hospitalizations between MI groups.

**Table 2:** Outcomes among 10,383 Myocardial Infarction Patients Discharged Alive.

Outcomes	Total	Anterior MI	Other MI	P-value
	N = 10,383	N = 2,942	N = 7,441	
**Ischemic Stroke**				
In-hospital ischemic stroke rate	20 (0.2%)	7 (0.2%)	13 (0.2%)	0.51
30-Day ischemic stroke rate	89 (0.9%)	30 (1.0%)	59 (0.8%)	0.26
90-Day ischemic stroke rate	143 (1.4%)	44 (1.5%)	99 (1.3%)	0.52
1-Year ischemic stroke rate	291 (2.8%)	87 (3.0%)	204 (2.7%)	0.55
4-Year ischemic stroke rate	577 (5.6%)	169 (5.7%)	408 (5.5%)	0.60
**Readmission for Bleeding**				
1-Year readmission for GI hemorrhage	162 (1.6%)	43 (1.5%)	119 (1.6%)	0.61
1-Year readmission for cerebral hemorrhage	20 (0.2%)	7 (0.2%)	13 (0.2%)	0.51
**Mortality**				
1-Year mortality	1,192 (11.5%)	347 (11.8%)	845 (11.4%)	0.53
4-Year mortality	2,514 (24.2%)	688 (23.4%)	1,826 (24.5%)	0.22

Data are reported as number (percentage) of patients or mean ± standard deviation (SD) value unless otherwise specified.

### Independent Risk Factors for Stroke

To determine risk factors associated with the occurrence of stroke after anterior MI and, in particular, whether longitudinal warfarin use had any significant protective effect on stroke occurrence, an analysis was restricted to those 1483 patients aged 66 and older, who were eligible for Ontario's universal drug benefit plan. Compared to the entire cohort of anterior MI patients, elderly patients were less frequently male (55 percent vs. 67 percent, P<0.001), more likely to have prior heart failure (7 percent vs. 4 percent, P<0.01) and hypertension (50 percent vs. 44 percent, P<0.01). Elderly anterior MI patients were less likely to be treated with immediate reperfusion therapy (38 percent vs. 48 percent P<0.001) and had a higher likelihood of developing in-hospital atrial fibrillation (8 percent vs. 6 percent P<0.001) as a complication of the index MI. Upon discharge from hospital, elderly anterior MI patients were more likely to be prescribed an ACE inhibitor (63 percent vs. 59 percent; P<0.01) and antiarrhythmic therapy or digoxin (18 percent vs. 12 percent; P<0.01), less likely to be prescribed a beta-blocker (63 percent vs. 69 percent; P<0.01) and statin (27 percent vs. 32 percent; P<0.01), with similar rates of anticoagulation and aspirin use at discharge.

The unadjusted characteristics of elderly patients, who developed and did not experience an ischemic stroke within 4 years after discharge for an anterior MI, are shown in Table 3. Patients who had a stroke after anterior MI were more likely to have previous diabetes mellitus (43 percent vs. 25 percent; P<0.001), higher systolic blood pressure upon discharge (126 mmHg vs. 120 mmHg; P = 0.006), develop atrial fibrillation (14 percent vs. 8 percent; P = 0.023) as a complication of their index anterior MI, and less likely to be prescribed an ACE inhibitor (53 percent vs. 63 percent; P = 0.02) and beta-blocker (53 percent vs. 64 percent; P = 0.02). There was no significant difference in the rate of warfarin use, at or after discharge, among patients who developed and did not experience an ischemic stroke after an anterior MI.

**Table 3:** Univariate Analysis of Four-Year Ischemic Stroke Risk among 1,483 Elderly Anterior Myocardial Infarction Patients.

Variable	Stroke	Non-Stroke	P-value
	N = 118	N = 1,365	
**Patient Demography**			
Age at admission for MI	76.77±6.69	76.52±6.97	0.71
Male gender	58 (49.2%)	756 (55.4%)	0.19
**Past Medical History**			
Diabetes mellitus	50 (42.4%)	346 (25.3%)	<.001
Myocardial infarction	33 (28.0%)	319 (23.4%)	0.26
Congestive heart failure	8 (6.8%)	92 (6.7%)	0.99
Hypertension	63 (53.4%)	677 (49.6%)	0.43
Previous smoker	32 (27.1%)	363 (26.6%)	0.90
Previous atrial fibrillation	2 (1.7%)	16 (1.2%)	0.62
Previous ischemic stroke	4 (3.4%)	22 (1.6%)	0.16
**In Hospital Findings & Procedures**			
Killip class ≥3	6 (5.1%)	51 (3.7%)	0.47
LV dysfunction	27 (22.9%)	334 (24.5%)	0.70
LV aneurysm, thrombus, or VSD	6 (5.1%)	67 (4.9%)	0.93
Reperfusion therapy	37 (31.4%)	525 (38.5%)	0.13
In-hospital onset of atrial fibrillation	16 (13.6%)	104 (7.6%)	0.02
In-hospital onset of ischemic stroke	0 (0.0%)	5 (0.4%)	0.51
Discharge systolic blood pressure	126.11±25.59	120.47±20.31	0.006
Discharge diastolic blood pressure	67.18±13.42	66.39±12.04	0.51
**Discharge Medications**			
ACE inhibitors	62 (52.5%)	868 (63.6%)	0.017
ARB	2 (1.7%)	41 (3.0%)	0.42
Antiarrhythmic therapy or digoxin	26 (22.0%)	239 (17.5%)	0.22
Aspirin	80 (67.8%)	987 (72.3%)	0.30
Beta-adrenergic antagonists	62 (52.5%)	868 (63.6%)	0.02
Calcium antagonists	35 (29.7%)	335 (24.5%)	0.22
Diuretics	42 (35.6%)	476 (34.9%)	0.88
LMWH	3 (2.5%)	24 (1.8%)	0.54
Nitrates	53 (44.9%)	580 (42.5%)	0.61
Clopidogrel	7 (5.9%)	64 (4.7%)	0.54
Statins	29 (24.6%)	372 (27.3%)	0.53
Warfarin	15 (12.7%)	205 (15.0%)	0.50
**Drug Benefit Claims for Warfarin**			
Within 30-days post discharge	11 (9.3%)	185 (13.6%)	0.19
Within 90-days post discharge	12 (10.2%)	191 (14.0%)	0.25

Data are reported as number (percentage) of patients or mean ± standard deviation (SD) value unless otherwise specified.

**Figure 1:**
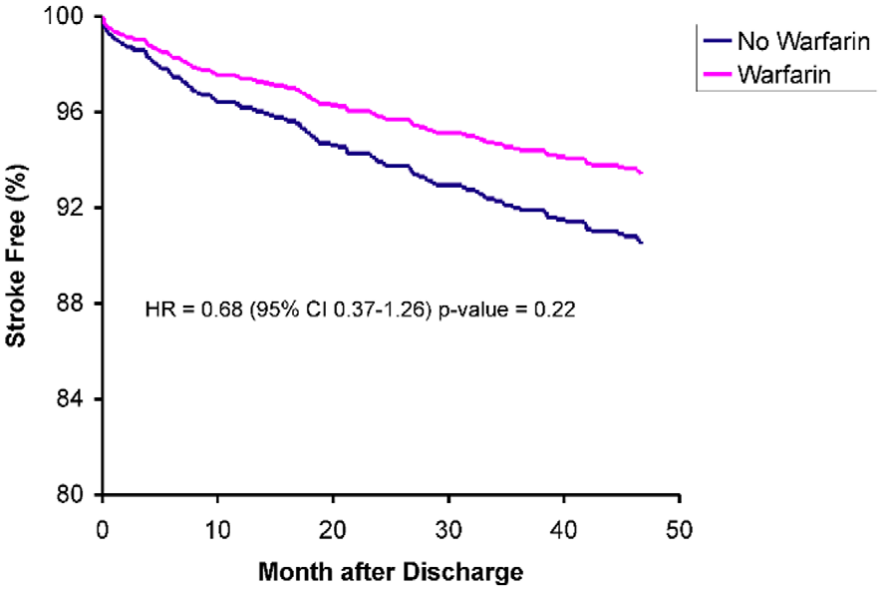
Adjusted Ischemic Stroke-Free Survival among 1,483 Elderly Patients with Anterior Myocardial Infarction. Survival curves are stratified by warfarin use for up to 90 consecutive days after an anterior MI. The curve in pink represents patients prescribed warfarin (patient received one or more prescriptions for warfarin after discharge). The curve in blue represents patients not prescribed warfarin.

Independent predictors of stroke after anterior MI were determined by multivariate analysis and are reported in Table 4. A previous history of diabetes mellitus (hazard ratio [HR], 2.35; 95% confidence interval [CI], 1.63–3.40) and receiving antiarrhythmic therapy or digoxin (HR, 1.60; 95% CI, 1.01–2.52) were independent predictors of stroke following anterior MI. Receiving a beta-blocker (HR, 0.60; 95% CI, 0.41–0.87) or an ACE inhibitor (HR, 0.65; 95% CI, 0.44–0.95) upon discharge for anterior MI were significant protective factors, but warfarin use for up to 90 days post-MI was not (HR, 0.68; 95% CI, 0.37–1.26). Thirty-seven percent of patients discharged on warfarin were also prescribed an antiarrhythmic agent or digoxin, representing a significant interaction (φ = 0.24, p<0.0001). The adjusted stroke-free survival analysis in patients surviving an anterior MI according to warfarin use is shown in Figure 1.

**Table 4:** Multivariate Model of Four-Year Ischemic Stroke Risk among 1,483 Elderly Patients with Anterior Myocardial Infarction.

Variable	Hazard Ratio (95% CI)	P-value
Warfarin user for 90 days	0.68 (0.37–1.26)	0.22
Previous diabetes mellitus	2.35 (1.63–3.40)	<.001
Discharged on antiarrhythmic therapy or digoxin	1.60 (1.01–2.52)	0.045
Discharged on an ACE inhibitor	0.65 (0.44–0.95)	0.025
Discharged on beta-adrenergic antagonists	0.60 (0.41–0.87)	0.008

Candidate variables were included in the initial Cox regression model if they were associated with stroke in a univariate analysis (P<0.25) listed in Table 3. Backward variable elimination, with an elimination criterion of a P value of more than 0.05, was then used to create a parsimonious model for predicting ischemic stroke.

Among the entire cohort who survived to hospital discharge, independent predictors of ischemic stroke after any type of MI were determined by univariate and multivariate analysis (Tables 5 and 6). Positive predictors of stroke after MI included older age, a previous history of hypertension, heart failure, diabetes, prior stroke, and LV dysfunction complicating the index MI. Coronary reperfusion therapy, discharge statin therapy, and aspirin were protective factors, while discharge diuretic therapy increased long-term stroke risk. The development of in-hospital atrial fibrillation and discharge on warfarin therapy were not independent predictors of long-term stroke after MI in this cohort.

**Table 5:** Characteristics of Patients Diagnosed with Ischemic Stroke among 10,383 Patients within Four Years of Hospital Discharge for Myocardial Infarction.

Variable	Total	Stroke	Non-Stroke	P-value
	N = 10,383	N = 577	N = 9,806	
**Patient Demographics**				
Age at admission of MI	66.35±13.52	73.01±10.32	65.96±13.59	<.001
Male gender	6,670 (64.2%)	307 (53.2%)	6,363 (64.9%)	<.001
**Past Medical History**				
Diabetes mellitus	2,576 (24.8%)	226 (39.2%)	2,350 (24.0%)	<.001
Myocardial infarction	2,273 (21.9%)	166 (28.8%)	2,107 (21.5%)	<.001
Congestive heart failure	410 (4.0%)	45 (7.8%)	365 (3.7%)	<.001
Hypertension	4,570 (44.0%)	319 (55.3%)	4,251 (43.4%)	<.001
Previous smoker	2,522 (24.3%)	144 (25.0%)	2,378 (24.3%)	0.73
Previous atrial fibrillation	118 (1.1%)	16 (2.8%)	102 (1.0%)	<.001
Previous ischemic stroke	131 (1.3%)	24 (4.2%)	107 (1.1%)	<.001
**In Hospital Findings & Procedures**				
Anterior wall myocardial infarction	2,942 (28.3%)	169 (29.3%)	2773 (28.2%)	0.60
Peak CK >2 times the upper limit of normal	6,148 (59.2%)	318 (55.1%)	5,830 (59.5%)	0.028
Killip class ≥3	239 (2.3%)	18 (3.1%)	221 (2.3%)	0.182
LV dysfunction	1,392 (13.4%)	104 (18.0%)	1,288 (13.1%)	<.001
LV aneurysm, thrombus, or VSD	214 (2.1%)	14 (2.4%)	200 (2.0%)	0.53
In-hospital onset of atrial fibrillation	588 (5.7%)	52 (9.0%)	536 (5.5%)	<.001
ST elevation on any lead groups	4,852 (46.7%)	236 (40.9%)	4,616 (47.1%)	0.003
Reperfusion therapy	3,300 (31.8%)	125 (21.7%)	3,175 (32.4%)	<.001
Discharge systolic blood pressure	120.38±19.62	125.12±22.90	120.11±19.38	<.001
Discharge diastolic blood pressure	67.88±11.62	68.64±12.50	67.84±11.57	0.12
**Discharge Medications**				
ACE Inhibitors	5,449 (52.5%)	319 (55.3%)	5,130 (52.3%)	0.187
ARB	213 (2.1%)	13 (2.3%)	200 (2.0%)	0.73
Antiarrhythmic therapy or digoxin	1,190 (11.5%)	105 (18.2%)	1,085 (11.1%)	<.001
Aspirin	7,734 (74.5%)	385 (66.7%)	7,349 (74.9%)	<.001
Beta-adrenergic antagonists	6,880 (66.3%)	337 (58.4%)	6,543 (66.7%)	<.001
Calcium antagonists	2,659 (25.6%)	167 (28.9%)	2,492 (25.4%)	0.065
Diuretics	2,510 (24.2%)	213 (36.9%)	2,297 (23.4%)	<.001
LMWH	232 (2.2%)	9 (1.6%)	223 (2.3%)	0.26
Nitrates	4,245 (40.9%)	271 (47.0%)	3,974 (40.5%)	0.003
Clopidogrel	511 (4.9%)	26 (4.5%)	485 (4.9%)	0.62
Statin	3,380 (32.6%)	131 (22.7%)	3,249 (33.1%)	<.001
Warfarin	850 (8.2%)	66 (11.4%)	784 (8.0%)	0.004

Data are reported as number (percentage) of patients or mean ± standard deviation (SD) value unless otherwise specified.

**Table 6:** Multivariate Model of Four-Year Ischemic Stroke Risk among 10,383 Myocardial Infarction Patients Discharged Alive.

Variable	Hazard Ratio (95% CI)	P-value
Discharge on warfarin	1.04 (0.78–1.37)	0.81
Age at admission of MI	1.04 (1.03–1.05)	<.001
Discharge diastolic blood pressure	1.01 (1.00–1.02)	0.03
Hypertension	1.21 (1.01–1.44)	0.04
Previous diabetes mellitus	1.82 (1.52–2.18)	<.001
Previous congestive heart failure	1.45 (1.04–2.02)	0.03
Previous ischemic stroke	2.38 (1.45–3.78)	<.001
Reperfusion therapy	0.80 (0.64–0.98)	0.04
LV dysfunction	1.27 (1.01–1.59)	0.04
Discharge on a statin	0.70 (0.57–0.86)	<.001
Discharge on a diuretic	1.23 (1.01–1.49)	0.04
Discharge on aspirin	0.73 (0.61–0.89)	0.002

Candidate variables were included in the initial Cox regression model if they were associated with stroke in a univariate analysis (P<0.25). Backward variable elimination, with an elimination criterion of a P value of more than 0.05, was then used to create a parsimonious model for predicting ischemic stroke.

## Discussion

Among elderly patients newly-hospitalized for an acute anterior-wall STEMI who survived to hospital discharge, we observed no significant reduction in ischemic stroke with warfarin use upon discharge or for a further 30-, 60-, or 90 days.

The utility of anticoagulation for the prevention of stroke after anterior MI is unclear. Anticoagulation might prevent the development of LV thrombus, which once detected is considered an established risk factor for embolic stroke [7], [14], [16]–[25]. However, previous estimates of anterior MI as an independent risk factor for LV thrombus or stroke have been inconsistent [5]–[14], [16]–[25], and information on their propensity for development after current standard interventions (e.g. antiplatelet and fibrinolytic therapy) for acute MI is limited [14]. Some studies have suggested that LV dysfunction is a stronger predictor of LV thrombus formation [16], [17], [21], [22] and the development of stroke [4]. Hence, the 2005 American College of Cardiology/American Heart Association practice guidelines [15] recommend those with STEMI complicated by LV dysfunction and extensive regional wall-motion abnormalities or LV mural thrombus noted on an imaging study receive a minimum of 3 months of warfarin for secondary prevention of stroke. There have only been a few small, non-blinded, randomized trials and observational studies of warfarin for established LV thrombus after MI for secondary prevention of systemic emboli, and these have demonstrated inconsistent results [36]–[39]. All were conducted before fibrinolytic and antiplatelet therapy had become routine. Our data suggests that overall, anterior MI patients do not have a higher risk of stroke, or death, as compared to other types of MI patients.

No prospective randomized controlled trial using warfarin has been conducted to study its efficacy after anterior STEMI in preventing left ventricular thrombus for prevention of stroke. The Fragmin in Acute Myocardial Infarction trial [40] prospectively demonstrated that administration of subcutaneous dalteparin (150 IU/kg body weight every 12 hours) for the duration of hospital stay after anterior MI reduced the risk of LV thrombus formation by 37 percent. But this trial was underpowered to demonstrate a significant effect on stroke and only followed patient outcomes for 11 days. Administration of subcutaneous heparin after thrombolysis for a STEMI [12], or alone in anterior STEMI [41], also did not demonstrate an effect on stroke. In a retrospective analysis [42], warfarin use in patients with heterogeneous causes of chronic left ventricular dysfunction demonstrated improved survival and risk of hospitalization for heart failure, but had no effect on stroke.

Meta-analyses of randomized controlled studies of moderate intensity warfarin with aspirin therapy after acute coronary syndrome (ACS) have demonstrated a relative risk reduction of stroke of approximately 40–46%, representing an absolute risk reduction between 0.5–1.28 percent [43], [44]. These studies were conducted before routine use of coronary artery stenting and, as warfarin is not considered adequate for preserving stent patency compared to thienopyridine agents [45], [46], [47], its use has lost ground to competing antiplatelet and antithrombin regimens. Warfarin use after a MI has not become the standard of practice within North America also due to concerns regarding its cost-effectiveness [48] and potential bleeding complications [49]. Stroke outcomes after MI may still change as practice patterns incorporate new guidelines based on studies that demonstrated benefit in combined cardiovascular endpoints with the use of various antithrombin agents [50], [51], [52] and newer antiplatelet agents [53], [54] throughout the index hospitalization after acute MI.

Patients who developed stroke after MI were older, had previous hypertension, heart failure, diabetes, prior stroke, and LV dysfunction complicating their index MI (Table 6), in keeping with well established risk factors for stroke following MI. These observations reinforce that secondary prevention efforts should focus on identifying and treating these risk factors for stroke, prior to hospital discharge as opposed to routine pre-emptive anticoagulation of all anterior MI patients. For instance, ACE inhibitor [55], [56], [57], [58] and beta-blocker [59], [60], [61], [62] therapy after acute MI complicated by congestive heart failure or LV dysfunction has demonstrated a 15–20% relative risk reduction in mortality and cardiovascular morbidity within stable patients. Whether a specific class of antihypertensive therapy offers additional protection against stroke post-MI, particularly in prevention of atrial fibrillation, has yet to be determined [63].

A major strength of our study was the longitudinal analysis of medication use and consideration of relative indications for appropriate anticoagulation after index hospitalization, such as atrial dysrhythmia. We also considered relative contraindications to, and complications from, anticoagulation therapy, such as major bleeding that required hospitalization. Our research has some limitations that merit emphasis. Our study was a population-based cohort analysis, and as such has inherent limitations and survival bias associated with its retrospective nature and lack of randomization. As well, the elderly cohort of anterior MI patients who survived to hospital discharge may not represent the general post-MI population. Because we used administrative data to measure stroke outcomes, we were limited in the amount of clinical information regarding the nature of each outcome. For instance, left ventricular ejection fraction data was only available for approximately half of the entire EFFECT cohort. We cannot ascertain data on the development of conditions that did not require hospitalization, such as out-of-hospital atrial fibrillation, stroke or hemorrhage, which may have occurred over the 4 year period, and as such our study may be underpowered due to the limited number of stroke hospitalizations. Ischemic stroke etiologies vary, and may be from causes other than cardioembolism, such as large artery atherosclerosis or small-vessel lacunar disease. In addition, we cannot ascertain the appropriateness of warfarin therapy, intensity of anticoagulation, or compliance in individual patients. For instance, our observation that a significant proportion of warfarin users who developed stroke were concomitantly discharged on an antiarrhythmic agent or digoxin, may suggest that many of these patients developed atrial dysrhythmia, a well-known immediate post-MI complication, and may be appropriately anticoagulated. Finally, we cannot exclude a short-term benefit from anticoagulation during the acute hospitalization phase post-MI as the number, and information on the timing, of stroke prior to discharge is limited.

In conclusion, among elderly patients who survive an anterior-wall MI, there may be no benefit from the routine use of warfarin up to 90 days post-MI in preventing ischemic stroke. Many practitioners still consider a large anterior-wall MI as high risk for potential LV thrombus formation and stroke. Our data would suggest that routine anticoagulation of patients with anterior-wall MI may not be indicated, although certain high-risk subgroups (e.g. documented LV thrombus or atrial fibrillation) may benefit from warfarin administration in this setting. Prospective randomized trials focusing on alternative antithrombin or antiplatelet strategies are needed to determine the best method for preventing this common and serious adverse outcome.

## Supporting Information

Table S1 International Classification of Diseases Codes.
(0.03 MB XLS)
